# (−)-Epigallocatechin-3-Gallate Inhibits the Life Cycle of Pseudorabies Virus *In Vitro* and Protects Mice Against Fatal Infection

**DOI:** 10.3389/fcimb.2020.616895

**Published:** 2021-01-14

**Authors:** Changchao Huan, Weiyin Xu, Tingting Guo, Haochun Pan, Hengyue Zou, Luyao Jiang, Chengmin Li, Song Gao

**Affiliations:** ^1^ College of Veterinary Medicine, Institutes of Agricultural Science and Technology Development, Yangzhou University, Yangzhou, China; ^2^ Jiangsu Co-Innovation Center for Prevention and Control of Important Animal Infectious Diseases and Zoonoses, Yangzhou, China; ^3^ Key Laboratory of Avian Bioproduct Development, Ministry of Agriculture and Rural Affairs, Yangzhou, China; ^4^ College of Medicine, Yangzhou University, Yangzhou, China; ^5^ Jiangsu Key Laboratory of Sericultural Biology and Biotechnology, School of Biotechnology, Jiangsu University of Science and Technology, Zhenjiang, China; ^6^ Institutes of Agricultural Science and Technology Development, Yangzhou University, Yangzhou, China

**Keywords:** (−)-epigallocatechin-3-gallate (EGCG), pseudorabies virus (PRV), antiviral, infection, adsorption, entry, replication

## Abstract

A newly emerged pseudorabies virus (PRV) variant with enhanced pathogenicity has been identiﬁed in many PRV-vaccinated swine in China since 2011. The PRV variant has caused great economic cost to the swine industry, and measures for the effective prevention and treatment of this PRV variant are still lacking. (–)-Epigallocatechin-3-gallate (EGCG) exhibits antiviral activity against diverse viruses and thus in this study, we investigated the anti-PRV activity of EGCG *in vitro* and *in vivo*. EGCG significantly inhibited infectivity of PRV Ra and PRV XJ5 strains in PK15 B6 cells and Vero cells. The anti-PRV activity of EGCG was dose-dependent, and 50 μM EGCG could completely block viral infection at different multiplicities of infection. We next revealed that EGCG blocked PRV adsorption and entry to PK15 B6 cells in a dose-dependent manner, but inhibition of PRV entry by EGCG was not as efficient as its inhibition of PRV adsorption. PRV replication was suppressed in PK15 B6 cells treated with EGCG post-infection. However, EGCG did not affect PRV assembly and could promote PRV release. Furthermore, 40 mg/kg EGCG provided 100% protection in BALB/c mice challenged with PRV XJ5, when EGCG was administrated both pre- and post-challenge. These results revealed that EGCG exhibits antiviral activity against PRV mainly by inhibiting virus adsorption, entry and replication *in vitro*. Meanwhile, EGCG increased the survival of mice challenged with PRV. Therefore, EGCG might be a potential antiviral agent against PRV infection.

## Introduction

Pseudorabies, a disease caused by pseudorabies virus (PRV) which infects both domestic and wild animals, is characterized by fever, severe itching (except in pigs), respiratory and nervous system disorders, and encephalomyelitis (Brittle et al., 2004). In the agricultural industry, it causes significant economic losses because it can lead to abortion or death of fetuses, weight loss in adult pigs, and death of newborn piglets (Pensaert, 1989; Klupp et al., 2004; Pomeranz et al., 2005; Szpara et al., 2011).

Since 2011, the prevalence of swine pseudorabies in Chinese farms has increased significantly. Further studies have found that the increasing incidence of pseudorabies may be related to insufficient protection stimulated by the traditional vaccine, Bartha-k61 (Wang et al., 2014; Gu et al., 2015). Furthermore, the new pseudorabies virus variant is different from the classic PRV strain. The variant disease is highly pathogenic in all infected pigs, and induces varying degrees of clinical symptoms, which means that existing vaccines only provide limited immune protection (An et al., 2013; Wu et al., 2013; Yu et al., 2014). In addition to infecting pigs, cattle, sheep, dogs, cats and other vertebrates, there have been reported mutations in the pig pseudorabies virus, which results in the infection of humans (Brittle et al., 2004; Blanchard et al., 2006; Ai et al., 2018). There is a report on PRV isolated from an acute human encephalitis case (Liu et al., 2020). Therefore, the prevention and control of PRV infection are particularly urgent.

(–)-Epigallocatechin-3-gallate (EGCG) is the most abundant bioactive polyphenol found in solid green tea extracts and has a variety of physiological and pharmacological effects on human health (McKay and Blumberg, 2002; Chacko et al., 2010). It has antibacterial, antiviral, antioxidant, antiarthritic, antiangiogenic, anti-inflammatory and antitumor activities (Haqqi et al., 1999; Osada et al., 2001; Sartippour et al., 2002; Dona et al., 2003; Weber et al., 2003; Sudano Roccaro et al., 2004). Studies have shown that EGCG has antiviral activity against retroviruses, orthomyxoviruses and flaviviruses *in vitro* (Fassina et al., 2002; Yamaguchi et al., 2002; Jariwalla et al., 2007; Roomi et al., 2008; Calland et al., 2012; Chen et al., 2012; Fukazawa et al., 2012). However, inhibitory effects of EGCG on pseudorabies virus (PRV) have not yet been reported. Therefore, we studied the antiviral effects of EGCG in PRV-infected host cells and mice. Our data demonstrated that EGCG significantly inhibited PRV infection at micromolar concentration, and EGCG could completely block PRV infection in the pig kidney cell line, PK15 B6, at 50 μM. Furthermore, EGCG inhibited PRV attachment, entry and replication in PK15 B6 cells. *In vivo* studies demonstrated antiviral activity of EGCG in BALB/c mice challenged intraperitoneally with a lethal dose of PRV. These results suggested that EGCG could be further developed as an antiviral agent against PRV infection.

## Materials and Methods

### Cells and Virus

PK15 B6 cells were separated from the porcine kidney cell line PK15 and were cultured in Dulbecco’s modified Eagle medium (DMEM) supplemented with penicillin and streptomycin, fungizone, and 4% fetal bovine serum(Lonsa S711-001). Vero cells were cultured in DMEM supplemented with penicillin, streptomycin, fungizone and 8% fetal bovine serum. All cells were cultured at 37°C with 5% CO_2_.

The virus strain, PRV XJ5, was isolated by the Yangzhou University Infectious Diseases laboratory and used in experiments. Virus stocks were stored at −80°C. PRV Ra was purchased from China Institute of Veterinary Drug Control.

### Reagents and Antibodies

(–)-Epigallocatechin-3-gallate (EGCG) (Selleck, China) was diluted to stock solutions of 50 mM with PBS and stored at −80°C for all subsequent experiments.

UL42, gE, and PRV-positive sera were generated in our laboratory. β-actin antibody was obtained from TransGenBiotech (Beijing, China). FITC-conjugated goat anti-pig IgG antibody was purchased from Sigma-Aldrich (St. Louis, MO). DAPI was purchased from Beyotime Biotechnology (Shanghai, China).

### Cytotoxicity Assay

The cytotoxicity of EGCG in PK15 B6 cells was assessed by CCK8 assay according to the manufacturer’s instructions. PK15 B6 cells were seeded in a 96-well plate at about 2×10^3^cells/well and cultured for 20 h. These cells were then treated with different concentrations (10, 20; or 50mM) of EGCG for 24 h, with six replicates for each concentration. After 24 h of culture, 10 μl of CCK-8 solution per well was added and the plate was further incubated for 2 h at 37°C. Absorbance was measured at 450 nm to find the maximum drug concentration that was not toxic to the cells.

### Cell-Based Assays

#### Infectivity Assay

PK15 B6 cells were pre-treated with different concentrations of EGCG (10, 20, or 50 μM) in DMEM at 37°C for 1 h. Next, PRV (multiplicity of infection [MOI]=0.1) was inoculated onto the cells. At 1 h post infection (hpi), the EGCG-virus supernatant was removed and cells were washed three times with PBS. The cells were then incubated with their corresponding concentrations of EGCG in DMEM containing 2% FBS (2% DMEM). At 24 hpi, intracellular viral proteins and DNA were detected by Western blot and qRT-PCR, respectively. Cell supernatants were used to measure virus titer.

#### (−)-Epigallocatechin-3-Gallate Pretreatment to Assess Pseudorabies Virus Adsorption and Entry

PK15 B6 cells were pretreated with 10, 20, or 50 μM EGCG at 37°C for 1 h. The cells were washed three times with cold PBS, then infected with PRV XJ5 (MOI=0.1) at 4°C for 1 h in the presence of corresponding concentrations of EGCG. Cells were washed with PBS three times and incubated in 2% DMEM containing appropriate concentrations of EGCG for 1 h at 37°C. The infected cells were washed with citric acid to remove extracellular bound virus and PBS three times respectively. At 24 hpi, intracellular viral proteins were detected by Western blot. Cell supernatants were used to measure virus titer by TCID_50_ assay.

PK15 B6 cells were treated as above, but at 1 hpi, the cells were washed with PBS and added in 1 ml DMEM to obtain cells for measuring virus DNA copies by quantitative (q) RT-PCR.

#### (−)-Epigallocatechin-3-Gallate Pretreatment to Assess Adsorption of Pseudorabies Virus

PK15 B6 cells were pretreated with 10, 20, or 50 μM EGCG for 1 h at 37°C. The cells were then washed with cold PBS three times, infected with PRV XJ5 (MOI=0.1) at 4°C and incubated with corresponding concentrations of EGCG for 1 h. The cells were washed three times with PBS and maintained in 2% DMEM in the absence of EGCG for 24 h. Intracellular viral proteins were detected by Western blot. Cell supernatants were used to measure virus titer by TCID_50_ assay.

PK15 B6 cells were treated as above, but at 1 hpi, the cells were washed with PBS and added in 1 ml DMEM to obtain cells for measuring virus DNA copies by quantitative (q) RT-PCR.

#### Post-Infection (−)-Epigallocatechin-3-Gallate Treatment to Assess Entry of Pseudorabies Virus

PK15 B6 cells were infected with PRV XJ5 at 4°C for 1 h without EGCG pre-treatment. The infected cells were washed with PBS three times and then cultured in 2% DMEM containing different concentrations of EGCG at 37°C for 1 h. Cells were washed three times with citric acid followed by three washes with PBS. Cells were then maintained in 2% DMEM without EGCG for 24 h at 37°C. Intracellular viral proteins were detected by Western blot. Cell supernatants were used to measure virus titer by TCID_50_ assay.

PK-15 B6 cells were treated as above, but after infection for 1 h, the cells were washed with citric acid and PBS three times respectively, then incubated with 1 ml DMEM to obtain cells for measuring virus DNA copies by qRT-PCR.

#### Replication, Assembly, and Release of Pseudorabies Virus

PK15 B6 cells were incubated with PRV XJ5 at 37°C for 1 h. The cells were then washed three times with PBS and incubated with 2% DMEM containing 10, 20, or 50 μM EGCG. At 2, 4, and 6 hpi, intracellular viral proteins were detected by Western blot. At 24 hpi, cells and supernatants were collected to determine the virus titer and viral DNA copies by TCID_50_ assay and qRT-PCR, respectively.

#### Western Blotting

Cell lysis buffer (Beyotime) was used to lyse the cells. Cell lysate proteins were mixed with 2× SDS-PAGE sample loading buffer and separated by 12% SDS-PAGE, then transferred onto a nitrocellulose membrane, which was blocked with 3% non-fat powdered milk in PBST for 2 h at room temperature. The membrane was then incubated with different primary antibodies at 4°C overnight. The next day, the membrane was incubated with the corresponding secondary antibodies at room temperature for 1.5 h. Immune complex bands were analyzed by the Super ECL Reagent Solution kit (Shanghai share Biotechnology, China).

### Virus Titer Assays

The 50% tissue culture infective dose (TCID_50_) assay was used to evaluate virus titers. Vero cells were seeded in 96-well plates with 8% FBS in DMEM. Virus samples were serially diluted from 10^-1^ to 10^-7^ in DMEM and used to inoculate cells at 37°C for 1.5 h, before removing the virus-DMEM mixture. The infected cells were cultured in 2% DMEM at 37°C with 5% CO_2_ for 72 h. Cytopathic effect (CPE) on Vero cells was counted to calculate the TCID_50_ by the Reed–Muench method.

#### DNA Extraction and qRT-PCR

Cell intracellular and extracellular viral DNA were extracted by the phenol chloride method (Zhou et al., 2017). In brief, 10% SDS and Proteinase K were added in samples and incubated at 56°C for 2h, followed by adding equal volume of phenol: chloroform (1:1, V/V) and vortexing, centrifuged for 15 min. The supernatant was collected and mixed with an equal volume of anhydrous ethanol, and kept at -20°C for 15min. The samples were centrifuged at 4°C for 10 min, and the precipitate was washed with 70% ethanol. The DNA was dissolved by ddH2O. PRV DNA was quantified by qRT-PCR with the following primers: gB94,5′-ACAAGTTCAAGGCCCACATCTAC-3′ (forward), gB94,5′-GTCCGTGAAGCGGTTCGTGAT-3′ (reverse). The reaction volume was 20 μl, with reaction conditions for fluorescence quantification of pre-denaturation at 95°C for 600 s, denaturation at 95°C for 10 s, and annealing at 62°C for 20 s for a total of 45 cycles.

#### Indirect Immunofluorescent Assay

PK15 B6 cells were ﬁxed with 4% paraformaldehyde for 15 min before incubation with 0.1% Triton X-100 for 15 min, and blocking with 5% BSA for 30 min. After blocking, the cells were incubated with PRV-positive pig serum at 37°C for 1 h and then with FITC-conjugated goat anti-pig IgG antibody at 37°C for 30 min. These cells were incubated with DAPI for 5 min before being observed under fluorescence microscopy. All images were taken at 100× magniﬁcation.

#### (−)-Epigallocatechin-3-Gallate Protective Efficacy Experiment in Mice

Six-week-old pathogen-free female BALB/c mice were purchased from the Experimental Animal Center at Yangzhou University (Yangzhou, China), with the Institutional Animal Care and Use Committee (IACUC) number: YZUDWLL-201910-001. The mice were divided into nine groups (each group has six mice), comprising PBS, virus-infected, EGCG control groups, EGCG low dose (20 mg/kg) 4 days prior, EGCG low dose 2 days prior, EGCG low dose 1 hpi, EGCG high dose (40 mg/kg) 4 days prior, EGCG high dose 2 days prior and EGCG high dose 1 hpi groups. For the EGCG-treated animals, EGCG was dissolved in PBS, and the mice were treated with a low dose (20mg/kg), or a high dose (40mg/kg) of EGCG by intraperitoneal injection at 4 days and 2 days before challenge with PRV XJ5 (10^4^ TCID_50_/ml) and 1 h after challenge with PRV XJ5 by intramuscular injection into the inner thigh, respectively. Each treatment group was daily administrated with EGCG for 4 days. The PBS and virus control groups were given a volume of PBS equal to the volume of EGCG received by the high dose group (40 mg/kg).

### Statistical Analysis

All experiments were independently repeated at least three times and all data were presented as means ± SD. The data were analyzed by GraphPad Prism software (GraphPad Software, San Diego, CA). When P <0.05, the differences were considered to be statistically significant.

## Results

### (−)-Epigallocatechin-3-Gallate Inhibits Pseudorabies Virus Infection in PK15 B6 Cells

**Figure 1:**
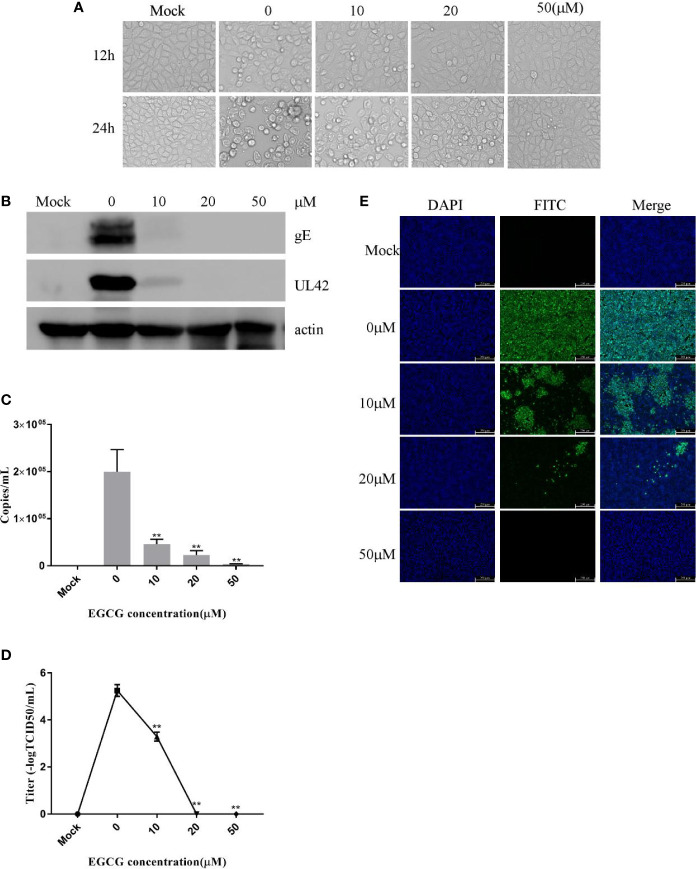
(–)-Epigallocatechin-3-gallate (EGCG) inhibited pseudorabies virus (PRV) infection in PK15 B6 cells. **(A)** Infected cells treated with 10, 20, or 50 μM EGCG for 12 or 24 h, showing changes in cell morphology. **(B)** PK15 B6 cells were pretreated with 10, 20, or 50 μM EGCG for 1 h before being infected with PRV XJ5 (MOI=0.1), then infected cells cultured with 10, 20, or 50 μM EGCG. At 24 hpi, gE, UL42 and β-actin proteins were detected by Western blot, **(C)** Viral DNA copy numbers were quantified by qRT-PCR. **(D)** Virus titer was evaluated by 50% tissue culture infective dose (TCID_50_) and **(E)** immunofluorescent assay (IFA)**** for internalized virus was performed. **p < 0.01.

EGCG did not cause signiﬁcant cytotoxic effects in PK15 B6 cells at concentrations up to 50 μM for 24 h (data not shown). To investigate the role of EGCG on PRV infection, PK15 B6 cells were pretreated with different concentrations of EGCG (10, 20, and 50 μM) for 1 h, and then infected with PRV XJ5 (MOI=0.1) in the presence of different concentrations EGCG for 24 h. **Figure 1A** shows that PRV XJ5 (MOI=0.1) caused obvious CPE in PK15 B6 cells, which could be inhibited by EGCG, especially at 50 μM EGCG. The antiviral effect of EGCG against PRV was further demonstrated by Western blot analysis. The data showed that PRV-gE and UL42 protein expression was reduced, and no viral protein expression was seen at concentrations of 20 and 50 μM EGCG (**Figure 1B**). qRT-PCR analysis showed that EGCG treatment resulted in an approximately 100% reduction of viral gB gene expression at a concentration of 50 μM. The inhibition of PRV infection by EGCG was dose-dependent, with the half-maximal response dose (EC_50_) being roughly 6.4 μM (**Figure 1C**). Furthermore, supernatant of infected PK15 B6 cells treated with EGCG were collected to determine the viral titer by TCID_50_. As expected, EGCG treatment reduced the production of virions, and no viral DNA was detectable in the supernatant in the presence of 20 and 50 μM EGCG (**Figure 1D**). Meanwhile, immunofluorescent assay (IFA) results confirmed that EGCG treatment significantly inhibited PRV infection in a dose-dependent manner (**Figure 1E**). These data indicated that EGCG inhibited PRV infection in PK15 B6 cells.

**Figure 2:**
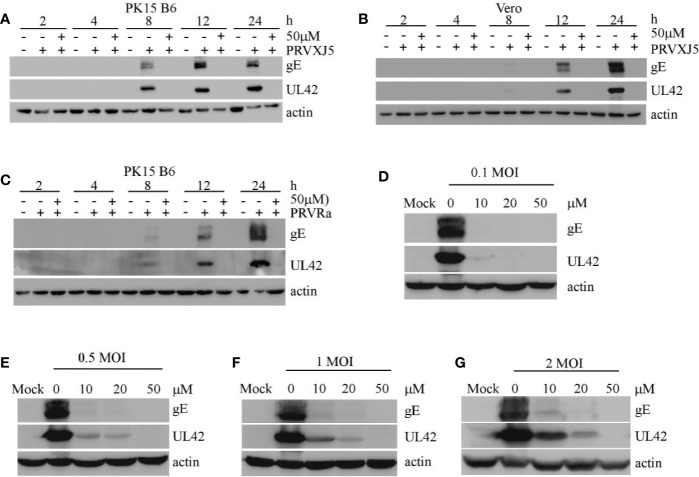
(–)-Epigallocatechin-3-gallate (EGCG) inhibited pseudorabies virus (PRV) infection activity at differing timepoints, for differing PRV strains and MOIs. **(A, B)** PK-15 and Vero cells were pretreated with 50 μM EGCG followed by infection with PRV XJ5 (MOI=0.1). At 2, 4, 8, 12, and 24 hpi, Western blot was used to evaluate PRV-gE, UL42 and β-actin protein expression. **(C)** PK15 B6 cells were pretreated with 50 μM EGCG followed by infection with PRV Ra (MOI=0.1). At 2, 4, 8, 12, and 24 hpi, Western blot was used to evaluate PRV-gE, UL42, and β-actin protein expression. **(D–G)** PK15 B6 cells were pre-treated with 10, 20, or 50 μM EGCG for 1 h before infection with PRV XJ5 at MOI=0.1, 0.2, 1, or 2, then cultured with 10, 20, or 50 μM EGCG. At 24 hpi, cell samples were collected and lysed for Western blot to analyze changes in gE, UL42 and β-actin protein expression.

To further investigate the effect of EGCG on PRV, PK15 B6 cells and Vero cells pretreated with 50 μM EGCG were infected with PRV XJ5 MOI=0.1. Western blotting indicated that at 2, 4, 8, 12, and 24 hpi, 50 μM EGCG could effectively inhibit PRV XJ5 infection (**Figures 2A, B**). Furthermore, Western blot analysis revealed that 50 μM EGCG could effectively inhibit PRV Ra infection at different times (**Figure 2C**). Subsequently, the cells were infected with different MOI (0.1, 0.5, 1, 2) of PRV XJ5 in the presence 20 or 50 μM EGCG for 24 h. The expression of PRV protein gE and UL42 was analyzed by Western blot (**Figures 2D–G**), which indicated that the antiviral effect of EGCG was greatest at low MOI of PRV, but 50 μM EGCG could completely block viral infection at different MOI.

### The Effect of (−)-Epigallocatechin-3-Gallate on Pseudorabies Virus Adsorption and Entry in PK15 B6 Cells

#### (−)-Epigallocatechin-3-Gallate Inhibits Pseudorabies Virus Adsorption and Entry to PK15 B6 Cells

**Figure 3:**
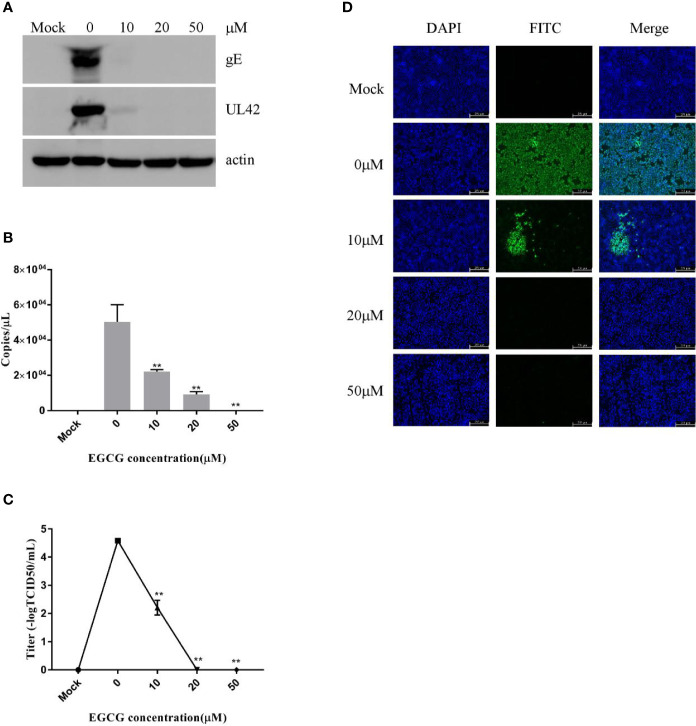
(–)-Epigallocatechin-3-gallate (EGCG) prevented pseudorabies virus (PRV) adsorption and entry to PK15 B6 cells. PK15 B6 cells were pretreated with 10, 20, or 50 μM EGCG for 1 h before cells were infected with PRV XJ5 (MOI=0.1) at 4℃ for 1 h and then incubated with 10, 20, or 50 μM EGCG at 37℃ for 1 h before removal of the EGCG medium and culture in EGCG-free DMEM. **(A)** At 24 hpi, Western blot detected PRV gE, UL42, and β-actin protein expression. **(B)** Intracellular virus DNA was quantified by qRT-PCR, **(C)** viral DNA titers were detected by 50% tissue culture infective dose (TCID_50_) and **(D)** immunofluorescent assay (IFA)**** for internalized virus was performed. **p < 0.01.

To clarify the effect of EGCG on virus adsorption and entry, PK15 B6 cells were pretreated with various concentrations of EGCG for 1 h before infection with PRV XJ5 (MOI=0.1) in the presence of various concentrations of EGCG for at 4°C for 1 h and then incubated with 10, 20, or 50 μM EGCG at 37°C for 1 h, before culture in 2% DMEM. The cells were collected at 24 hpi. Expression levels of PRV gE and UL42 protein were reduced, especially in the presence of 20 and 50 μM EGCG (**Figure 3A**). Meanwhile, the supernatant was collected and viral titer was determined at 24 hpi, indicating that the PRV viral titer was decreased signiﬁcantly in cells treated with EGCG (**Figure 3C**). Furthermore, to confirm the effect of EGCG on PRV adsorption and entry, viral copy numbers in the cells were determined at 1 hpi, which demonstrated viral copies in cells treated with EGCG were signiﬁcantly impaired in a dose-dependent manner (**Figure 3B**), with an EC_50_ of approximately 9.6 μM. Together, these results confirmed that EGCG inhibited virus adsorption and entry in a dose-dependent manner, with 50 μM EGCG completely inhibiting PRV infection. Additionally, IFA assay further demonstrated that EGCG treatment prevented virion attachment and entry to cells in a dose-dependent manner (**Figure 3D**).

#### (−)-Epigallocatechin-3-Gallate Affects Pseudorabies Virus Adsorption, Entry Into PK15 B6 Cells Respectively

**Figure 4:**
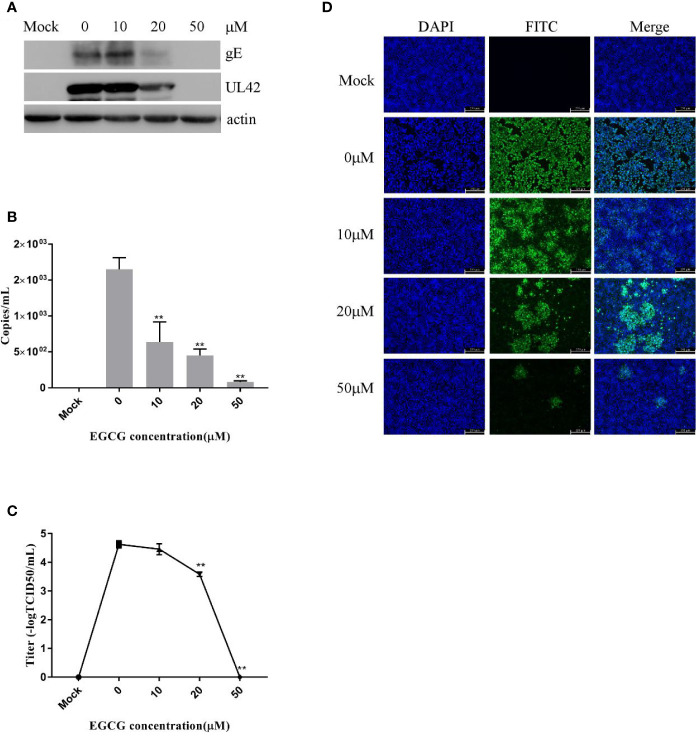
(–)-Epigallocatechin-3-gallate (EGCG) prevents pseudorabies virus (PRV) adsorption in PK15 B6 cells. PK15 B6 cells were pre-treated with 10, 20, or 50 μM EGCG for 1 h, then infected with PRV XJ5 (MOI=0.1) at 4℃ for 1 h in the presence of 10, 20, or 50 μM EGCG before removal of the EGCG medium and culture in EGCG-free Dulbecco’s modified Eagle medium (DMEM). **(A)** At 24 hpi, PRV gE, UL42 and β-actin protein expression was analyzed by Western blot. **(B)** Viral DNA was quantified by qRT-PCR, **(C)** 50% tissue culture infective dose (TCID_50_) was used to detect viral titer and **(D)** immunofluorescent assay (IFA) for internalized virus was performed. **p < 0.01.

To investigate the effect of EGCG on virus adsorption, PK15 B6 cells were pretreated with various concentrations of EGCG for 1 h before infection with PRV XJ5 (MOI=0.1) in the presence of various concentrations of EGCG for 1 h at 4°C. The cells were then washed with PBS before incubation at 37°C. Cells were collected at 24 hpi, and Western blot revealed that PRV gE and UL42 protein expression levels were reduced in a dose-dependent manner (**Figure 4A**). To further assess the effect of EGCG on PRV adsorption, various concentrations of EGCG were incubated with the cells for 1 h before PRV infection for 1 h at 4°C. The EGCG-virus supernatant was removed. Intracellular viral DNA copies were analyzed by qRT-PCR at 24 hpi. As expected, EGCG effectively prevented virus adsorption (**Figure 4B**), with an EC_50_ of approximately 9.8 μM. Furthermore, similar dose-dependent inhibition of virus adsorption was observed by TCID_50_ assay in the cell supernatant (**Figure 4C**) and IFA assay (**Figure 4D**). The results indicated that EGCG blocked virus attachment to PK15 B6 cells.

**Figure 5:**
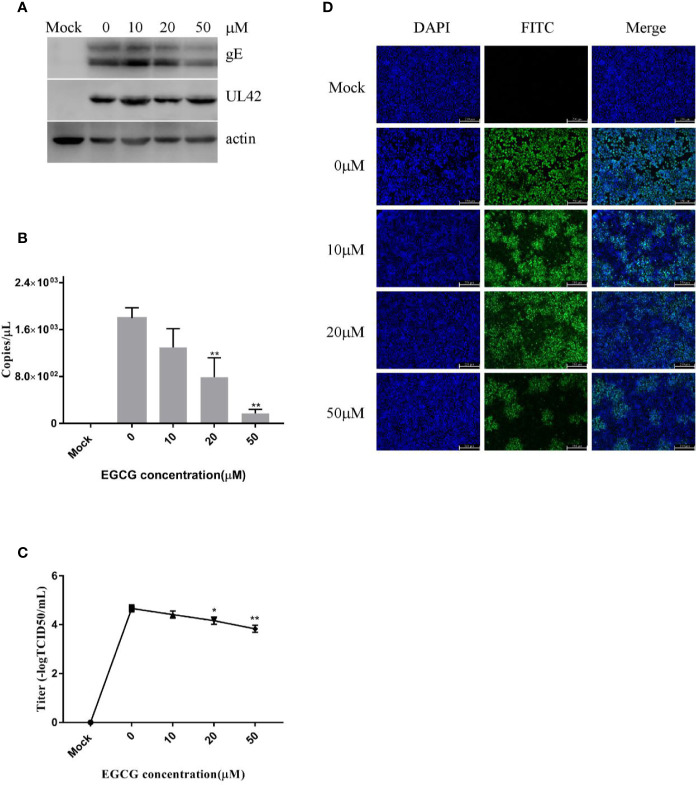
(–)-Epigallocatechin-3-gallate (EGCG) prevented pseudorabies virus (PRV) entry to PK15 B6 cells. PK15 B6 cells were infected with PRV XJ5 (MOI=0.1) at 4°C for 1 h, then infected cells were treated with 10, 20, or 50 μM EGCG for 1 h, before removal of the EGCG medium and culture in EGCG-free DMEM. **(A)** At 24 hpi, PRV gE, UL42, and β-actin protein expression were quantified by Western blot. **(B)** PRV gB DNA was quantified by qRT-PCR, **(C)** virus titer was evaluated by 50% tissue culture infective dose (TCID_50_) and **(D)** immunofluorescent assay (IFA) for internalized virus was performed. **p < 0.01.

To assess viral entry, PK15 B6 cells were treated with different concentrations of EGCG during the virus entry stage, without prior EGCG pre-treatment. The results confirmed that EGCG inhibited PRV entry in a dose-dependent manner (**Figures 5A–D**) with an EC_50_ of approximately 17.9 μM. However, inhibition of PRV entry by EGCG was not as significant as the observed inhibition of PRV adsorption.

Taken together, these data reveal that EGCG inhibited PRV adsorption and/or entry, but EGCG had a more significant inhibitory effect on PRV adsorption.

#### (−)-Epigallocatechin-3-Gallate Inhibits Pseudorabies Virus Replication in PK15 B6 Cells

**Figure 6:**
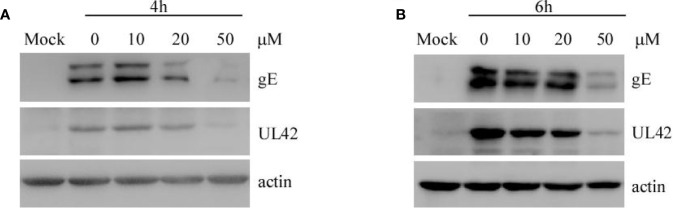
(–)-Epigallocatechin-3-gallate (EGCG) inhibited pseudorabies virus (PRV) replication in PK15 B6 cells. **(A, B)** PK15 B6 cells were infected with PRV XJ5 (MOI=0.1) at 37℃ for 1 h, then infected cells were incubated with 10, 20, or 50 μM EGCG until harvest. At 4 and 6 hpi, cells were collected to detect the expression of PRV-gE, UL42, and β-actin protein by Western blot.

To explore whether EGCG affects the replication of PRV in PK-15 B6 cells, cells were infected with PRV XJ5 (MOI=0.1) for 1 h, then treated with 10, 20, or 50 μM EGCG. The expression of virus protein gE and UL42 was analyzed by Western blot at 4 and 6 hpi. The results showed that EGCG reduced the expression of gE and UL42 in a dose-dependent manner (**Figures 6A, B**), indicating that EGCG could prevent virus replication.

#### (–)-Epigallocatechin-3-Gallate Affects Pseudorabies Virus Assembly and Release in PK15 B6 Cells

**Figure 7:**
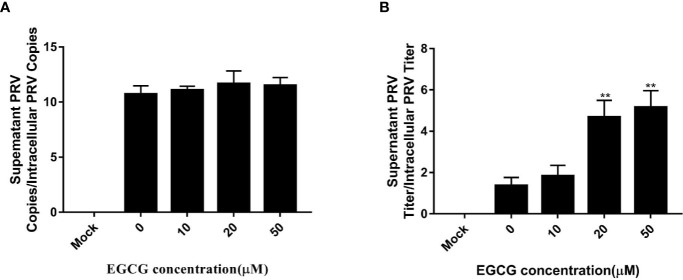
Effect of (–)-Epigallocatechin-3-gallate (EGCG) on pseudorabies virus (PRV) assembly and release. PK15 B6 cells were infected with PRV XJ5 (MOI=0.1) in 37°C for 1 h, then treated with 10, 20, 50, or 100 µM EGCG. **(A)** Supernatant and cells were harvested to determine PRV DNA copy numbers at 24 hpi and the ratio of extracellular to intracellular PRV DNA copies was calculated. **(B)** PRV viral titers in supernatant and cells were assayed at 24 hpi and the ratio of extracellular to intracellular PRV viral titers was calculated. **p < 0.01.

To assess whether EGCG affected virus assembly, cells were infected with PRV XJ5 (MOI=0.1) at 37°C for 1 h before incubation with 10, 20, 50, and 100 µM EGCG until harvest. The supernatant and the cells were collected to determine PRV DNA copy number at 24 hpi. The ratio of extracellular to intracellular PRV DNA copies revealed that EGCG had no effect on PRV assembly (**Figure 7A**). Simultaneously, the collected supernatant and cells were used to determine PRV viral titer and the ratio of extracellular to intracellular PRV viral titers was determined, which confirmed that EGCG might promote PRV release (**Figure 7B**).

### *In Vivo* Evaluation of the Antiviral Activity of (–)-Epigallocatechin-3-Gallate

**Figure 8:**
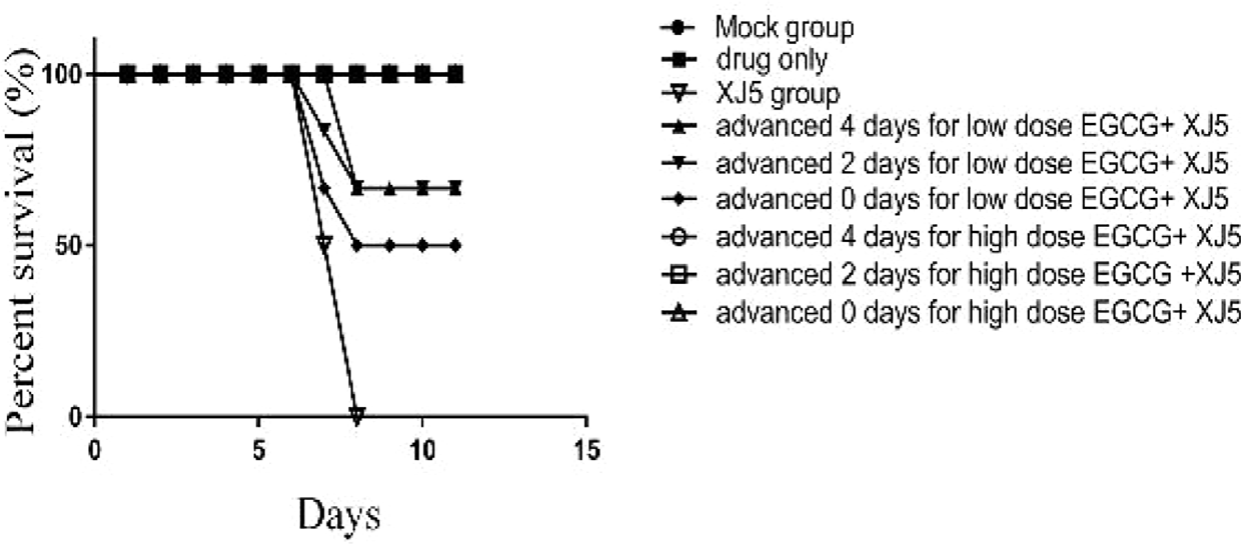
The antiviral effect of (–)-Epigallocatechin-3-gallate (EGCG) was evaluated in BALB/c mice divided into nine groups, control, PRV XJ5, EGCG, and EGCG+PRVXJ5 groups 20 or 40 mg/kg EGCG, injected at 4 or 2 days prior to challenge with PRV XJ5, or 1 h after challenge with PRV XJ5. The survival data for each group were shown.

We next investigated whether EGCG could protect BALB/c mice challenged with 10^4^ TCID_50_/ml PRV XJ5. BALB/c mice were administrated with EGCG (20 or 40 mg/kg, low and high dose groups, respectively) by intraperitoneal injection (ip) at 4 or 2 days prior to challenge with PRV XJ5 (10^4^ TCID_50_/ml), or at 1 h after challenge. Each treatment group was continuously administered with EGCG for 4 days. PBS- and PRV XJ5 infection control mice were given a volume of PBS equal to the volume of EGCG received by the high dose EGCG group. The mice in the challenge-only group showed pruritus, neurological symptoms and began to die on the third day post-challenge. Some mice in the low-dose EGCG inoculation group showed pruritus and neurological symptoms on the third day and died on the fourth day post-challenge. The mice administrated with high dose EGCG stayed healthy. The survival rates of the treated mice were shown in **Figure 8**. The mice in the PRV XJ5 infection group began to die on day 3. The survival rate of mice treated with 20 mg/kg EGCG ip at 4 or 2 days before challenge was 66.66%, while the survival rate of mice treated with 20 mg/kg EGCG ip at 1 h after challenge was 50%. Treatment with 40 mg/kg EGCG provided 100% protection at all times of administration. These results indicated that EGCG as a potential antiviral drug demonstrates therapeutic effect on PRV infection *in vivo*.

## Discussion

EGCG is a polyphenolic compound found in green tea extracts and possesses many beneficial properties, including antiviral activities (Xu et al., 2017). Previous reports have shown that EGCG inhibits virus-infected cells through different pathways; for example, it can inhibit the attachment of HSV-1 to heparin sulfate by interacting with virion surface proteins (Colpitts and Schang, 2014). It can also interact with HIV envelope glycoprotein to prevent viral entry into target cells (Liu et al., 2005). Furthermore, EGCG inhibits HCV replication by downregulating COX-2 (Lin et al., 2013).

The present study confirms that EGCG inhibits PRV infection during multiple steps of the virus life cycle. EGCG significantly inhibits PRV XJ5 and PRV Ra infection *in vitro*, mainly by inhibiting PRV adsorption, entry and replication. Furthermore, EGCG could reduce PRV attachment to PK15 B6 cells. Previous reports also indicated that there were two ways to inhibit virus infection in cells. Firstly, EGCG binds to target cells to block the adsorption of virus. Secondly, EGCG binds to virus to block virus attachment to target cells (Nakayama et al., 1993; Yamaguchi et al., 2002; Liu et al., 2005; Colpitts and Schang, 2014). We found that EGCG inhibited PRV infection through decreasing attachment to cells, but the mechanism requires further study.

EGCG prevents the infection of many viruses, including HCV, HIV-1, and HSV, from entering target cells (Yamaguchi et al., 2002; Weber et al., 2003; Isaacs et al., 2008). We also confirmed that EGCG decreased PRV entry to PK15 B6 cells. For PRV, EGCG inhibits PRV entry to PK15 B6, but this is less efficient than the observed inhibition of absorption.

Previous reports have suggested that EGCG treatment could inhibit virus replication. For example, EGCG inhibits HBV-induced incomplete autophagy to reduce HBV replication (Zhong et al., 2015); EGCG interferes with HCV replication by down regulating a COX-2 inhibitor (Lin et al., 2013). Furthermore, EGCG could suppress DNA and RNA synthesis steps to prevent virus replication (Chen et al., 2012; Pang et al., 2014; Karamese et al., 2015). Our data revealed that EGCG also inhibited PRV replication.

In our study, we found that EGCG could block PRV attachment, entry and replication *in vitro*. In mice, after the infection with PRV XJ5, different doses of EGCG and different times of ip administration were investigated. We found that the rates of protection were dose-dependent and the time of administration was important at the lower dose of EGCG. The results revealed that 20 mg/kg EGCG by ip injection at 4 days or 2 days prior to challenge with PRV XJ5 provided 66.66% protection, while 20 mg/kg EGCG by ip injection 1 h after challenge with PRV XJ5 provided 50% protection. However, 40 mg/kg EGCG provided 100% protection at all times of administration. These results demonstrated that 40 mg/kg EGCG provided complete protection to BALB/c mice challenged with a lethal dose PRV XJ5.

In conclusion, we have demonstrated here that EGCG inhibits PRV infection by interfering with PRV attachment, entry, and replication *in vitro*, particularly at the attachment stage. EGCG could prevent PRV assembly, but promote PRV release. Furthermore, 40 mg/kg EGCG administered to mice either before or shortly after lethal challenge with PRV was fully protective. These results reveal that EGCG could be further developed as an antiviral agent against PRV infection.

## Data Availability Statement

The original contributions presented in the study are included in the article/supplementary materials; further inquiries can be directed to the corresponding author.

## Ethics Statement

The animal study was reviewed and approved by Yangzhou university animal center.

## Author Contributions

CH and WX contributed equally to this work. All authors contributed to the article and approved the submitted version.

## Funding

This research was funded by the National Natural Science Foundation of China (31902253), the Natural Science Foundation of Jiangsu Province (BK20180921), the individual technology research and development of modern agricultural industry of Jiangsu Province (CX(19)3024), the China Postdoctoral Science Foundation (2018M632399), the Priority Academic Program Development of Jiangsu Higher Education Institutions (PAPD), and the earmarked fund for Jiangsu Agricultural Industry Technology System.

## Conflict of Interest

The authors declare that the research was conducted in the absence of any commercial or financial relationships that could be construed as a potential conflict of interest.
